# A retrospective impact analysis of the WannaCry cyberattack on the NHS

**DOI:** 10.1038/s41746-019-0161-6

**Published:** 2019-10-02

**Authors:** S. Ghafur, S. Kristensen, K. Honeyford, G. Martin, A. Darzi, P. Aylin

**Affiliations:** 1 NIHR Patient Safety Translational Research Centre, Imperial College London, London, UK; 2 Dr Foster Unit, Department of Primary Care and Public Health, Imperial College London, London, UK

**Keywords:** Health policy, Policy

## Abstract

A systematic analysis of Hospital Episodes Statistics (HES) data was done to determine the effects of the 2017 WannaCry attack on the National Health Service (NHS) by identifying the missed appointments, deaths, and fiscal costs attributable to the ransomware attack. The main outcomes measured were**:** outpatient appointments cancelled, elective and emergency admissions to hospitals, accident and emergency (A&E) attendances, and deaths in A&E. Compared with the baseline, there was no significant difference in the total activity across all trusts during the week of the WannaCry attack. Trusts had 1% more emergency admissions and 1% fewer A&E attendances per day during the WannaCry week compared with baseline. Hospitals directly infected with the ransomware, however, had significantly fewer emergency and elective admissions: a decrease of about 6% in total admissions per infected hospital per day was observed, with 4% fewer emergency admissions and 9% fewer elective admissions. No difference in mortality was noted. The total economic value of the lower activity at the infected trusts during this time was £5.9 m including £4 m in lost inpatient admissions, £0.6 m from lost A&E activity, and £1.3 m from cancelled outpatient appointments. Among hospitals infected with WannaCry ransomware, there was a significant decrease in the number of attendances and admissions, which corresponded to £5.9 m in lost hospital activity. There was no increase in mortality reported, though this is a crude measure of patient harm. Further work is needed to appreciate the impact of a cyberattack or IT failure on care delivery and patient safety.

## Introduction

The global ransomware attack, WannaCry, took hold across multiple continents and organisations on Friday 12 May, 2017.^1^ Although not directly targeted, one of the biggest causalities of this attack was the National Health Service (NHS) in England.^1^ Over 600 organisations were affected; this included 34 infected hospital trusts (NHS organisations that provide acute care, specialised medical services, mental healthcare, or ambulance services) and 46 affected hospital trusts.^1^ Infected hospital trusts were locked out of their digital systems and medical devices, such as MRI scanners; affected trusts were those that were not infected but reported disruption either through preventative action or sharing systems with infected organisations. The UK Department of Health and Social Care (DHSC) was alerted about the emerging events at 1 p.m. that day and by 4 p.m. a major incident was declared as the scale of the problem became more apparent.^1^ The attack was brought to a halt on the evening of the 12 of May by a cyber researcher who had activated a kill switch, which stops the spread of the malicious software, and prevented further devices from being infected.^1^ Over the next week, the cyberattack resulted in significant disruption across the NHS for patients and healthcare staff, which included reverting to manual processes (e.g.: reporting blood results, paper notes); disruption to radiology services; cancelled outpatient appointments, elective admissions, and day case procedures; and for five infected acute trusts, emergency ambulances were diverted to other hospitals.^2^

Cyber security attacks are a growing threat to healthcare and there have been a number of significant cyber security incidents in healthcare globally, the biggest being at Anthem Blue Cross Insurance System in the U.S., where over 78 million (m) health records were stolen in 2015.^3^ Most recently, the Singapore Health System reported a major breach of over 1 m patient records, including the prime minister’s record.^4^ Despite the number of reported cyberattacks on healthcare internationally, there has been no comprehensive assessment of the actual impact of any attack in terms of service disruption, financial impact, and harm to patients. Healthcare is one of the sectors most exposed to cyberattacks; this is partly because of the vulnerability of the systems, often running on legacy platforms.^5^ Medical records consist of financial information, health details, and social security information and are more in demand on the dark web than are credit card data.^6^ Despite the number of reported cyberattacks on healthcare internationally, there is paucity of information on the actual impact of any attack in terms of service disruption, financial impact, and harm to patients.

As healthcare systems across the world become increasingly dependent on digital systems to deliver care, it is crucial to understand the impact of any cyber security breach/attack on the functionality of the system and how we can improve digital resilience. This paper aims to provide a more in-depth review of the impact of the WannaCry ransomware attack on the NHS in England; however, the lessons drawn have a global resonance. The analysis has been made possible using Hospital Episodes Statistics (HES) to determine the number of cancelled outpatient appointments, the impact on emergency and elective admissions, the number of accident and emergency (A&E) attendances, deaths, and the financial impact on activity.

## Results

### Impact of WannaCry on hospital activity

Table 1 shows the total counts of activity across all trusts in the weeks before, during, and after the WannaCry attack. For all types of activity except outpatient cancellations, compared to the week before the attack, activity was lower during the WannaCry week. Activity tends, however, to fluctuate across the weeks displayed, and there is nothing to suggest that activity during the WannaCry week was abnormally low compared with other weeks.

**Table 1:** National activity counts in the weeks before, during, and after WannaCry

	Week					Total
	−2	−1	WannaCry week	+1	+2	April–June
Total admissions	273,727	303,386	297,840	302,986	265,193	3,755,086
Emergency admissions	142,485	145,178	144,492	146,547	140,759	1,854,462
Elective admissions	131,242	158,208	153,348	156,439	124,434	1,900,624
Day case admissions	108,395	130,281	126,141	128,613	102,994	1,565,867
Elective admissions excl. day cases	22,847	27,927	27,207	27,826	21,440	334,757
A&E attendances	373,542	374,710	365,833	371,676	375,949	4,806,543
Deaths in A&E	340	360	310	303	339	4218
Outpatient appointments	1,878,032	2,323,146	2,272,223	2,272,220	1,704,802	27,449,176
Outpatient attendances	1,485,163	1,836,566	1,779,498	1,786,203	1,336,314	21,539,339
Outpatient cancellations	132,541	164,408	175,552	163,215	126,517	2,050,352

*A&E* accident and emergency

At a hospital trust level, compared with the baseline, there was no statistically significant difference in the total level of activity across all trusts during the week of the WannaCry attack (Table 2). Hospital trusts had on average 1% more emergency admissions (1.1 admissions, 95% confidence interval 0.2 to 1.9) per day during the WannaCry week compared with baseline weeks, though compared to an average of 107 emergency admissions per trust per day, this is not a clinically significant increase in activity, and activity was also higher than the baseline period in the weeks before and after WannaCry, so this is unlikely to be related to the attack. There was also <1% fewer A&E attendances per hospital per day during the WannaCry week compared to the baseline weeks (−3.2 attendances, −5.3 to −1.2), but again, this difference is not clinically significant, and with similar volatility observed in the weeks preceding and after the WannaCry week, this difference is unlikely to be related with the WannaCry attack.

**Table 2:** Activity before, during, and after WannaCry across all trusts

	Total admissions	Emergency admissions	Elective admissions	Day case admissions	Elective admissions excl. day cases	A&E attendances	Deaths in A&E	Outpatient appointments	Outpatient attendances	Outpatient cancellations
Panel A: Point estimates and confidence intervals of difference in average daily activity per hospital in the weeks before, during, and after WannaCry across all trusts compared to baseline period										
−2	5.5	3	3.5	4.3	0.6	8.4	0	46	44.6	−3.6
	[1.9, 9.0]	[2.1, 3.8]	[0.4, 6.6]	[1.6, 7.0]	[−0.2, 1.3]	[6.3, 10.4]	[−0.0, 0.1]	[2.7, 89.3]	[11.5, 77.7]	[−10.6, 3.4]
−1	4.8	1.6	4.1	3.6	0.8	5.1	0	70.9	68.9	−3.5
	[1.4, 8.3]	[0.7, 2.4]	[1.1, 7.1]	[1.1, 6.2]	[0.1, 1.5]	[3.1, 7.2]	[0.0, 0.1]	[28.3, 113.4]	[36.4, 101.3]	[−10.3, 3.4]
WannaCry week	0.2	1.1	−0.3	−0.5	0.2	−3.2	0	26.3	21.1	4.4
	[−3.3, 3.7]	[0.2, 1.9]	[−3.3, 2.8]	[−3.0, 2.1]	[−0.5, 0.9]	[−5.3, −1.2]	[−0.0, 0.0]	[−16.3, 68.9]	[−11.4, 53.6]	[−2.5, 11.2]
+1	4.4	2.6	2.5	2.1	0.7	2.3	0	34.3	32.3	−4.2
	[1.0, 7.9]	[1.8, 3.5]	[−0.5, 5.5]	[−0.5, 4.6]	[0.0, 1.4]	[0.2, 4.3]	[−0.1, 0.0]	[−8.2, 76.9]	[−0.2, 64.8]	[−11.1, 2.7]
+2	−0.4	1.7	−2.3	−1.6	−0.8	10.6	0	−93.3	−74.8	−8.6
	[−3.9, 3.1]	[0.8, 2.5]	[−5.4, 0.8]	[−4.2, 1.1]	[−1.5, −0.1]	[8.6, 12.7]	[−0.0, 0.1]	[−136.8, −49.9]	[−108.0, −41.6]	[−15.6, −1.6]
* N*	17,882	17,299	15,790	13,096	15,070	13,832	13,832	17,114	17,114	17,114
Mean per trust per day	210	107.2	120.4	119.6	22.2	347.5	0.3	1603.9	1258.6	119.8
Panel B: Expected national activity during with and without WannaCry										
Predicted activity	296,718.9	143,798	151,903.5	126,568	26,964.8	365,833	310	2,285,402.3	1,790,122.8	176,435.8
Predicted activity if WannaCry week was similar to baseline week	296,449.1	142,379.4	152,208	127,038.4	26,765.1	369,256.9	320.6	2,250,567.3	1,762,225.9	170,669.2
Estimated difference	269.8	1418.6	−304.5	−470.4	199.6	−3423.9	−10.6	34,834.9	27,896.8	5766.6
	[−4489, 5028.7]	[317.3, 2519.9]	[−3996.6, 3387.6]	[−3097, 2156.2]	[−611.8, 1011.1]	[−5592.3, −1255.5]	[−48, 26.8]	[−21525.2, 91195.1]	[−15100.2, 70893.9]	[−3316.6, 14849.9]

Panel A: The dependent variable is activity per trust per day. Point estimates reflect the average difference in daily activity across all hospitals in weeks before, during, and after WannaCry compared to the baseline, which is any other day between 1 April and 30 June 2017. Regression controls for day of week, bank holiday, and hospital fixed effects. 95% confidence intervals in squared brackets. Panel B: Expected activity is the predicted activity from the regression

*A&E* accident and emergency

**Fig. 1:**
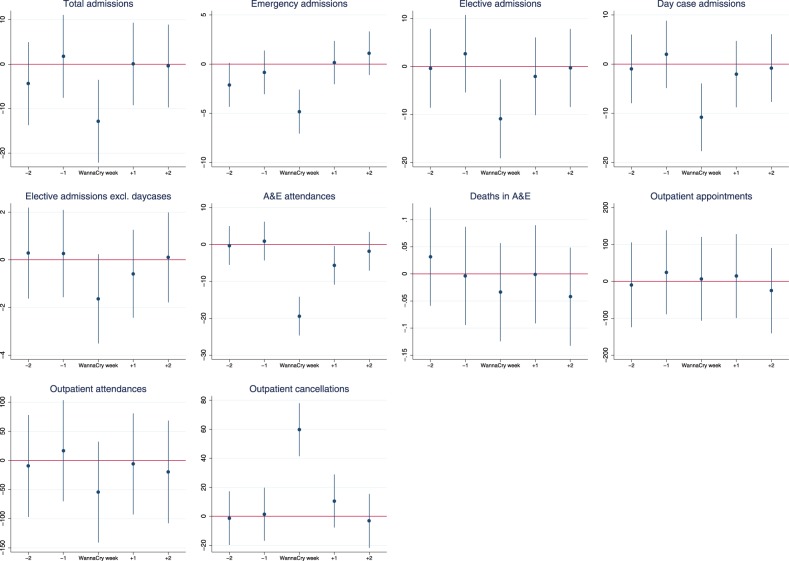
Difference in mean daily activity between infected and non-infected hospitals before, during, and after the WannaCry week. Point estimates and 95% confidence intervals for difference in mean daily activity between infected and non-infected hospitals during the WannaCry week

However, comparing infected to non-infected trusts, there was a statistically and clinically significant difference in activity levels at infected trusts during WannaCry, which was not observed in the weeks before or after the attack (Fig. 1 and Appendix Table 1). There was a decrease of about 6% in total admissions per infected hospital per day during WannaCry (−12.8 admissions, 95% confidence interval −22.1 to −3.5), with 4% fewer emergency admissions (−4.8, −7.1 to −2.6) and 9% fewer elective admissions (−10.9, −19.1 to −2.7). The decrease in elective admissions was driven by a decrease in day case admissions of 10.8 fewer admissions per hospital per day during WannaCry (−17.7 to −3.9), while there was no statistically significant difference in the number of elective admissions who were inpatients.

A&E departments were also affected, and there were on average 6% fewer attendances per infected hospital per day during WannaCry (−19.4 A&E attendances, 95% confidence interval −24.6 to −14.2). The decrease in A&E attendances at infected trusts lasted into the week after WannaCry, which saw on average 5.6 (−10.9 to −0.4) fewer A&E attendances per day at infected trusts compared to the baseline period.

The attack also affected outpatient services. During the WannaCry week, infected trusts had on average 50% more cancellations than non-infected trusts per day (59.7 cancellations, 95% confidence interval 41.4 to 78.0). This resulted in 55 fewer outpatient attendances per day at infected trusts, but this was not precisely estimated (−140 to 30.2).

### Mortality

Across all trusts, compared to the baseline week, there was no significant difference in the number of deaths in A&E. There was also no significant difference in deaths in A&E between infected and non-infected trust (0 deaths (−0.1 to 0.1).

### Financial impact

The total economic value of the lower activity at the infected trusts during the WannaCry week was £5.9 m (95% confidence interval £3.6 m to £8.2 m), including £4 m (£1.5 m to £6.6 m) in lost inpatient admissions, £0.6 m (£0.4 m to £0.8 m) from lost A&E activity, and £1.3 m (£0.9 m to £1.7 m) from cancelled outpatient appointments (Table 3). Assuming that all trusts had been infected by WannaCry and affected to the same extend as the actually infected trusts, the total value of lost activity could have amounted to £35 m (£21.2 m to £48.8 m) in activity alone.

**Table 3:** Estimated impact of WannaCry on total activity during the WannaCry week

	At actually infected trust		If all trusts were infected	
	Activity difference	Costed difference	Activity difference	Costed difference
Total admissions	−2935.6	−£4.0 m	−17,562.1	−£24 m
	[−5067.2, −803.9]	[−£6.6 m, −£1.5 m]	[−30,314.8, −4809.3]	[−£39.3 m, −£8.8 m
Emergency admissions	−1066	−£2.1 m	−6386.6	−£12.6 m
	[−1558.5, −573.5]	[−£3.1 m, −£1.1 m]	[−9337.1, −3436.1]	[−£18.4 m, −£6.8 m]
Elective admissions	−2175.6	−£1.9 m	−13,162.2	−£11.5 m
	[−3815.9, −535.3]	[−£3.5 m, −£0.3 m]	[−23,086.1, −3238.3]	[−£20.9 m, −£2.0 m]
Day case admissions	−1857.7	−£1.2 m	−11,016.4	−£7.2 m
	[−3038.6, −676.7]	[−£2.0 m, −£0.4 m]	[−18,019.6, −4013.1]	[−£11.8 m, −£2.6 m]
Elective admissions excl. day cases	−315.8	−£0.7 m	−1907.7	−£4.2 m
	[−676.6, 45.1]	[−£1.5 m, £0.1 m]	[−4087.9, 272.4]	[−£9.1 m, £0.6 m]
A&E attendances	−3760.2	−£0.6 m	−20,648.6	−£3.3 m
	[−4781.7, −2738.7]	[−£0.8 m, −£0.4 m]	[−26,224.6, −15,072.6]	[−£4.1 m, −£2.4 m]
Outpatient appointments	3328.8	£0.2 m	9303.7	£0.9. m
	[−21,730.7, 28,388.3]	[−£2.3 m, £2.6 m]	[−140,860.5, 159,467.9]	[−£13.7 m, £15.5 m]
Outpatient attendances	−12,166.8	−£1.2 m	−71,860.0	−£7.0 m
	[−31,562.4, 7228.8]	[−£3.1 m, £0.7 m]	[−186,415.1, 42,695.2]	[−£18.2 m, £4.2 m
Outpatient cancellations	13,534.4	£1.3 m	78,962	£7.7 m
	[9453.3, 17,615.4]	[£0.9 m, £1.7 m]	[54,791.4, 103,132.6]	[£5.3 m, £10.1 m]
Total financial impact		−£5.9 m		−£35.0 m
		[−£8.2 m, −£3.6 m]		[−£48.8 m, −£21.2 m]

Impact of WannaCry on activity calculated as the difference-in-differences estimate for difference in activity multiplied by the number of infected trusts. 95% confidence intervals in square brackets

*A&E* accident and emergency, *m* million

## Discussion

Our analysis of the HES data demonstrated the impact of the WannaCry attack across the NHS in England. This resulted in a 6% decrease in admissions in the infected hospitals, which included 1100 fewer emergency department (ED) admissions and 2200 fewer elective admissions in total. The infected hospitals also saw a decrease in the number of ED attendances with 3800 fewer patients seen. There was a significant impact on the number of outpatient cancellations across the infected hospitals during the WannaCry week—this resulted in 13,500 appointments being cancelled. The financial impact of the attack was also calculated, and the value of the reduction in the activity in the infected trusts amounted to £5.9 m. If this pattern were seen across all NHS hospitals, the reduced activity alone would have cost £35 m.

This is the first comprehensive analysis of this cyberattack across secondary care, both in terms of activity and economic impact. The National Audit Office Report and the Lessons learned review of the WannaCry ransomware cyber attack are the most in-depth analyses to date; however, they fail to fully explore the true impact of the attack across the English NHS.^1,2^ The reports describe the number of outpatient appointments that were cancelled: 19,000 in total but did not describe the impact on emergency or elective admissions or A&E attendances.

It was fortuitous that the kill switch was found on the same day as the attack happened; this somewhat limited the potential impact and threat to the health service.^1^ The numbers of patients who had to travel further were resorbed into the system, there was no increase in admissions overall, and the system demonstrated resilience and the ability to cope with changing pressures. News channels and social media reported extensively on the attack, and this may have contributed to the pattern seen as patients were able to see which hospitals were most affected.^1^ Yet, the resulting impact on patients and staff is not fully appreciated. Five hospitals, including Barts Health (Royal London Hospital), one of the main trauma centres in London, had to close their EDs; patients and emergency ambulances had to travel further to other hospitals to seek care; this had further impact on these hospitals in terms of increased numbers.^1^

Our analysis suggests that, if all hospitals had been infected, there would be 21,000 fewer ED attendances in total. While the system managed to resorb the number of patients during WannaCry, if the impact had been greater, we have no understanding on the network effect of what would happen and what the contingency plans would be. Depending on the scale of the attack and the reliance of the organisation on information technology (IT) systems in the delivery of care, disruption may range from inconvenience for the clinical workforce, with little or no discernible impact on patient care, to a complete shutdown of clinical service provision. To understand this better, we are carrying out further research to predict the redistribution of emergency care demand in the context of hospital closures to ensure neighbouring centres are adequately prepared in case of such an event.

A significant 13,500 outpatient appointments in the infected hospitals had to be cancelled. NHS England identified that there were at least 139 cancellations for patients with potential cancer, who were referred to urgent clinics.^2^ It is difficult to appreciate the full impact of these cancellations on patient care, as we do not know for how long all of these appointments were further delayed, and the cascade effect on patients at a time when patients were already waiting longer for treatment.^7^

We found no significant effect demonstrated on mortality across all hospitals. This pattern was also the finding from a previous study using the same methodology on the impact on mortality during the junior doctors’ strikes that took place in England in 2016. Furnivall et al. reported on the impact of the strikes on patient morality and suggested that potential reasons for seeing this could be that their study did not have enough power to demonstrate an appreciable effect.^8^ Yet, they and others reporting on similar events also proposed that, during a period of stress for the service, staffing priority is often given to acute, emergency, and critical care services, and senior medical and nursing staff are often drafted into these areas to ensure the flow of care.^9^

The NAO stated that there were no reports of patient harm from NHS organisations.^1^ This is difficult to quantify, and as discussed, mortality is a crude measure of patient harm. While the attack may not have led to a direct impact on mortality, we are unable to ascertain the true impact on complications, patient morbidity, or changes in care processes that resulted from the attack.

Because of the complexity of any healthcare system, it is understandably difficult to fully appreciate the impact of any cyberattack. Yet, any impact on a given system can and will undermine the safety of patients. Published examples of the effects resulting from IT failures, often seen in cyberattacks, include the loss of access to electronic health records and radiology and pathology results, drug dosing and drug administration errors, lack of contingency planning when traditional work patterns are affected, and, in the worst-case scenario, patient deaths due to incorrect data.^10–12^

As we become ever more reliant on digital services to deliver healthcare on a global level, it is crucial to fully appreciate the implications of IT flaws and failings to mitigate any harm to patients. To further appreciate disruptions in care delivery and how we actually measure the impact on patient safety in the event of IT failure/cyberattacks, we are carrying out qualitative interviews with staff from the infected Trusts.

We carried out our analysis of the financial impact of WannaCry at aggregate level, based on the tariffs for outpatient appointments, day case and elective admissions, and ED attendances and admissions. This assessment is an estimate based on the information collected by the DHSC and resulted in a total of £5.9 m based on lost activity to NHS Trusts. We also calculated that, if all NHS Trusts in England had been infected on that day, the resulting costs, based on tariffs for different activities, would be £35 m. The opportunity cost is significant and this total does not account for the additional costs that would be required for IT support to restore and recover systems.

A recent report published by the DHSC has estimated that the cost to the NHS during the attack was approximately £19 m because of lost output and a further £0.5 m for additional IT support.^13^ The report also factored in a further £73 m on further IT support required to recover data and restore systems.^13^ Most infected Trusts were unable to estimate the financial impact of the attack on their organisation, though Barts Health NHS Trust reported that their estimate was approximately £4.8 m, which included loss of income and hiring of digital experts to support the recovery process post attack.^13^ The DHSC’s estimate was based on an anticipation that WannaCry would disrupt 1% of all NHS services including primary care, whereas our estimate is based on actually observed changes in activity, but only considers secondary care.

A study by IBM and the Ponemon Institute reported that cyber breaches in the US cost up to $6.2 billion per year and that almost 90% of hospitals have reported a data breach.^13^ Costs that have been accounted for are not just the obvious damage to digital networks and systems, loss in revenue, or data theft but include others such as costs of reporting, legal action against the organisation, the cost of reputational damage, and fines from national bodies for any data breaches.^14^ Again, because of complexity of healthcare as a sector, it is often difficult to estimate a true and comprehensive cost of any cyberattack.^15^

As a sector, healthcare is one of the most vulnerable to cyberattacks, yet it has chronically underinvested in cyber resilience.^5^ Since WannaCry, there has been a considerable increase in capital investment to shore up cybersecurity for the NHS, though with the current scale and threat of the problem, alongside the investment, there needs to be an increase in IT budgets to ensure that current systems can be sustained securely and that healthcare systems are resilient in the face of attack.^5^

The NAO report stated that none of the organisations affected by WannaCry had followed advice by NHS Digital (the national information and technology partner to the health and social care system) to apply a Microsoft update patch, which resulted in the vulnerability being exposed.^1^ This highlights the legacy systems and infrastructure that are in use, and since the WannaCry attack, funding has been made available for NHS organisations to upgrade their software to Microsoft Windows 10 to improve resilience.^16^ This also raises the issue of education, awareness, and sharing of information to ensure that a national learning system exists and good practice can be spread.^17^ NHS Digital collects information on cyber threats and the impact from any breaches. These are disseminated across the NHS through the CareCERT bulletins.^1^ The above example highlights, however, that more needs to be done in terms of shared learning, information sharing, education, and leadership both at the national and local levels.^14^

To prevent or mitigate these types of events from recurring in the NHS or in any other healthcare organisation, there is a need to develop and test effective incident management procedures and improve business continuity planning.^18,19^ All organisations must be able to safely and effectively function while under cyberattack. Meanwhile, all data and systems must be securely backed-up and disaster recovery processes tested to ensure that the backup is isolated and cannot be erased or tampered with.^18^ Strong leadership and a security culture throughout the healthcare sector can help significantly to improve patient safety.^18^

The weaknesses of the study are predominantly due to what was not investigated/captured. Because of the data set used, this study does not capture the resulting impact on primary care services, and the NAO report and subsequent follow-up reports by DHSC and NHS England have not detailed the full impact on primary care or social care.

It is difficult to capture the true impact of the cyberattack, as mortality is a crude measure of patient harm and there is no current way to quantify patient harm, lapses, and patient safety. If computer systems were down, staff would also be unable to report any patient safety incidents that would otherwise be reported using the NRLS. This is also true for the recording of any data/events during the WannaCry period. If systems to code and collect administrative data were down, the data held by DHSC and NHS England may not accurately reflect the full extent of events.

Using the national-level data, this study has demonstrated the impact of a cyberattack on a healthcare system. Healthcare has become one of the most vulnerable sectors to cyberattacks globally. Although not targeted at the NHS directly, the WannaCry attack had a significant negative impact on the delivery of care and cost to the health service in England. It was fortuitous that the attack was stopped within 24 h, though the impact on the service lasted longer, with significant numbers of outpatient appointments and elective and day case admissions being cancelled.

In the infected hospitals, there was also a significant decrease in the number of attendances and admissions, with five hospitals having to divert emergency care. There was no increase in mortality reported, though this is a crude measure of patient harm. As the health sector becomes ever more reliant on IT to deliver patient care, there needs to be adequate investment of resources and contingency plans in place to minimise harm and disruption to patients. These lessons resonate globally as we become ever more reliant on IT systems to help deliver healthcare. Further work needs to be done to appreciate the impact on care delivery and how we actually measure the impact on patient safety in the event of IT failure or cyberattack.

## Methods

### Study design

The principal investigator received approval from the Secretary of State and the Health Research Authority under Regulation 5 of the Health Service (Control of Patient Information) Regulations 2002 to hold confidential data and analyse them for research purposes (CAG ref. 15/CAG/0005). We have approval to use them for research and measuring quality of delivery of healthcare from the London - South East Ethics Committee (REC ref. 15/LO/0824).

### Data

HES includes details of all admissions, outpatient appointments, and attendances at EDs in all NHS hospitals in England and is collected by the Department of Health.^20^ In line with previous work,^7^ admitted patients were separated into elective and emergency categories using the “admimeth” method of admission field in HES. Outpatient appointments recorded as “seen” or “arrived late, but seen” in the “attended” field were counted as “attended”, and those that were “cancelled or postponed by the healthcare provider” were counted as cancelled.

### Period of study

The WannaCry attack occurred on the afternoon of Friday, 12 May.^1^ Data were extracted for all infected and non-infected trusts for the period 1 April to 30 June 2017 and aggregated to the hospital and day level.

### Trusts

We included all NHS trusts with >500 admissions or outpatient attendances across the study period in the analysis, including acute hospitals, community centres, and mental health trusts.

Trusts infected by the WannaCry virus were identified by the Department of Health. Thirty-four trusts were infected with the WannaCry virus, 36 trusts were affected, and 131 trusts were neither infected nor affected.^1^

### Outcomes

Outcomes were outpatient appointments cancelled, elective and emergency admissions to hospital, A&E attendances, and deaths in A&E.

### Analysis

We calculated activity totals for each of the outcomes in the weeks before, during, and after the WannaCry attack. We defined the week of the WannaCry attack as the 7 days after and including the first day of the attack (Friday, 12 May) and defined the 2 weeks before and the 2 weeks after the attack similarly. As the attack started on a Friday, the weeks before and after are also defined as 7 days starting from a Friday, rather than conventional weeks starting on a Sunday/Monday.

In order to determine the overall impact of the WannaCry attack on national activity, we estimated a model comparing average activity per trust per day during the WannaCry week and the 4 weeks surrounding the week of the attack to activity during the baseline period, which was any other week between 1 April and 30 June 2017. To understand the impact of WannaCry on total national activity, we compared predicted activity from our model to predictions of total national activity if activity during the WannaCry week had been similar to the baseline weeks. The estimated coefficients thus reflect the average difference in daily activity across all hospitals in weeks before, during, and after WannaCry compared to the baseline. We included dummy variables for day of week, bank holiday, and hospital fixed effects.

To examine the impact on activity specifically at the infected trusts, we compared the change in each outcome at the infected hospitals to the change in those outcomes at the non-infected hospitals in a difference-in-differences approach using ordinary least squares. In all models, we included control variables for day of the week and bank holidays and used hospital fixed effects to control for unobserved time invariant differences between hospitals. We also tested the difference in activity between hospitals that were affected and those neither affected nor infected.

When estimating the total impact on infected hospitals, we predicted the expected activity if the WannaCry week had been similar to the baseline weeks at the infected hospitals and compared the estimate of total activity to the actual activity at the infected trusts. We also calculated the expected impact if all hospitals had been infected and calculated the difference between actual and expected activity under this scenario.

We calculated the financial impact of WannaCry at actually and potentially infected hospitals by multiplying the total activity impact estimates with average tariffs for the specific type of activity. For inpatient and outpatient activities, we used activity weighted average tariffs, and for A&E activity, where activity data were not available, we used the average tariff. For A&E visits, it was £158, for emergency admissions £1970, day case admissions £655, elective admissions £2,222, and outpatient appointments £97.50.

### Reporting summary

Further information on research design is available in the Nature Research Reporting Summary linked to this article.

## Supplementary information

Supplementary table

Reporting summary

## Data Availability

Relevant data are available by application to NHS Digital.

## Supplementary information

**Supplementary information** accompanies the paper on the *npj Digital Medicine* website (10.1038/s41746-019-0161-6).

## Code availability

The relevant code is available from the corresponding author upon request.
