# Supplementary material for: A retrospective impact analysis of the WannaCry cyberattack on the NHS
Source: NPJ Digit Med. 2019 Oct 2;2:98. doi: 10.1038/s41746-019-0161-6 (PMC6775064; doi:10.1038/s41746-019-0161-6)
Supplement: Supplementary file 1 — Supplementary table [file 41746_2019_161_MOESM1_ESM.pdf]

Supplementary Table 1: Difference in differences estimates of the impact of WannaCry on hospitals at infected versus non-infected hospitals

	Total admissions	Emergency admissions	Elective admissions	Day case admissions	Elective admissions excl. day cases	A&E attendances	Deaths in A&E	Outpatient appointments	Outpatient attendances	Outpatient cancellations
Infected x 2 weeks before	-4.4	-2.1	-0.4	-1	0.3	-0.3	0	-9.2	-9.5	-1.3
	[-13.7,5.0]	[-4.3,0.1]	[-8.6,7.9]	[-7.9,6.0]	[-1.6,2.2]	[-5.5,5.0]	[-0.1,0.1]	[-124.0,105.6]	[-97.1,78.0]	[-19.7,17.2]
Infected x 1 week before	1.8	-0.8	2.7	2	0.3	0.9	0	24.7	16.7	1.5
	[-7.5,11.1]	[-3.1,1.4]	[-5.4,10.8]	[-4.9,8.8]	[-1.6,2.1]	[-4.3,6.2]	[-0.1,0.1]	[-88.9,138.4]	[-70.0,103.4]	[-16.8,19.7]
Infected x Wannacry week	-12.8	-4.8	-10.9	-10.8	-1.6	-19.4	0	7.0	-54.3	59.7
	[-22.1,-3.5]	[-7.1,-2.6]	[-19.1,-2.7]	[-17.7,-3.9]	[-3.5,0.2]	[-24.6,-14.2]	[-0.1,0.1]	[-106.5,120.5]	[-140.9,32.3]	[41.4,78.0]
Infected x 1 week after	0.1	0.2	-2	-2	-0.6	-5.6	0	14.3	-5.9	10.6
	[-9.2,9.3]	[-2.0,2.4]	[-10.1,6.1]	[-8.8,4.7]	[-2.4,1.3]	[-10.9,-0.4]	[-0.1,0.1]	[-99.4,128.0]	[-92.6,80.9]	[-7.7,28.9]
Infected x 2 weeks after	-0.4	1.1	-0.3	-0.8	0.1	-1.8	0	-25.1	-19.6	-3.1
	[-9.7,8.9]	[-1.1,3.3]	[-8.4,7.8]	[-7.7,6.1]	[-1.8,2.0]	[-7.1,3.4]	[-0.1,0.0]	[-140.6,90.3]	[-107.7,68.4]	[-21.7,15.4]
N	17882	17299	15790	13096	15070	13832	13832	17114	17114	17114

The coefficients are the estimated differences in average daily activity per hospital, controlling for day of the week, bank holidays, main effects of the weeks during, and two weeks before and after WannaCry as well as hospital fixed effects.
